# Novel CFD modeling approaches to assessing urine flow in prostatic urethra after transurethral surgery

**DOI:** 10.1038/s41598-020-79505-6

**Published:** 2021-01-12

**Authors:** Bin Zhang, Shuang Liu, Yinxia Liu, Bo Wu, Xuhui Zhang, Xin Wang, Xuezhi Liang, Xiaoming Cao, Dongwen Wang, Chin-Lee Wu

**Affiliations:** 1 Department of Urology, First Hospital of Shanxi Medical University, Taiyuan, 030001 Shanxi China; 2 Department of Urology, Massachusetts General Hospital, Harvard Medical School, Boston, MA 02114 USA; 3 Department of Obstetrics and Gynecology, Shanxi Health Vocational College, Shanxi Traditional Chinese Medicine School, Jinzhong, 030600 Shanxi China; 4 National Cancer Center/National Clinical Research Center for Cancer/Cancer Hospital & Shenzhen Hospital, Chinese Academy of Medical Sciences and Peking Union Medical College, No. 113 Baohe road, Longgang district, Shenzhen, 518116 China; 5 First College of Clinical Medicine, Shanxi Medical University, Taiyuan, 030001 Shanxi China; 6 Department of Pathology, Massachusetts General Hospital, Harvard Medical School, Boston, MA 02114 USA

**Keywords:** Urethra, Prostate, Benign prostatic hyperplasia, Urinary tract obstruction

## Abstract

Assessment of the pressure and velocity of urine flow for different diameter ratios of prostatic urethra (RPU) after transurethral surgery using computational fluid dynamics (CFD). A standardized and idealized two-dimensional CFD model after transurethral surgery (CATS-1st) was developed for post-surgery mid-voiding. Using CATS-1st, 210 examples were amplified according to an array of size [3][5][14], which contained three groups of longitudinal diameters of prostatic urethra (LD-PU). Each of these groups contained five subgroups of transverse diameters of the bladder neck (TD-BN), each with 14 examples of transverse diameters of PU (TD-PU). The pressure and velocity of urine flow were monitored through flow dynamics simulation, and the relationship among RPU-1 (TD-PU/TD-BN), RPU-2 (RPU-1/LD-PU), the transverse diameter of the vortex, and the midpoint velocity of the external urethral orifice (MV-EUO) was determined. A total of 210 CATS examples, including CATS-1st examples, were analyzed. High (bladder and PU) and medium/low (the rest of the urethra) pressure zones, and low (bladder), medium (PU), and high (the rest of the urethra) velocity zones were determined. The rapid changes in the velocity were concentrated in and around the PU. Laminar flow was present in all the examples. The vortices appeared and then gradually shrank with reducing RPU on both the sides of PU in 182 examples. In the vortex examples, minimum RPU-1 and RPU-2 reached close to the values of 0.79 and 0.02, respectively. MV-EUO increased gradually with decreasing RPU. In comparison to the vortex examples, the non-vortex examples exhibited a significantly higher (p < 0.01) MV-EUO. The developed CFD models (CATS) presented an effective simulation of urine flow behavior within the PU after transurethral surgery for benign prostatic hyperplasia (BPH). These models could prove to be useful for morphological repair in PU after transurethral surgery.

## Introduction

Morphological deformation of prostatic urethra (PU) is a significant factor contributing to benign prostatic hyperplasia (BPH), which ultimately leads to voiding dysfunction in elderly males. The deformation impedes the hydrodynamics of the urinary tract by causing urethral obstruction during micturition^1^. It is also the main link behind the post-surgery improvement in the urination function^2^. Recent studies have demonstrated that the vortex, recognized for its fluid energy loss^2^, occurs in the obstructed PU during spontaneous voiding in preoperative patients^3^, as well as in the completely-hollowed PU during emptying using equipment just after the surgery for BPH^4^. It follows that expansion, one of the forms of deformation, may also affect urine flow in a manner similar to obstruction. Therefore, it is necessary to understand the relationship between urine flow and luminal hollowing in PU. The common non-invasive measurement techniques, including ultrasound^5^ and magnetic resonance imaging (MRI)^6^, fail to provide sufficient information regarding the dynamic state of urine flow in the urethra^7^. Even, the traditional urodynamics tools encounter challenges in directly evaluating the hydrodynamic effects in vivo^8,9^.

Therefore, in the present study, computational fluid dynamics (CFD) modeling focusing on PU was employed to determine the pressure and velocity of urine flow with different diameter ratios of PU (RPU) mid-voiding after transurethral surgery.

## Materials and methods

### Measurements

**Figure 1:**
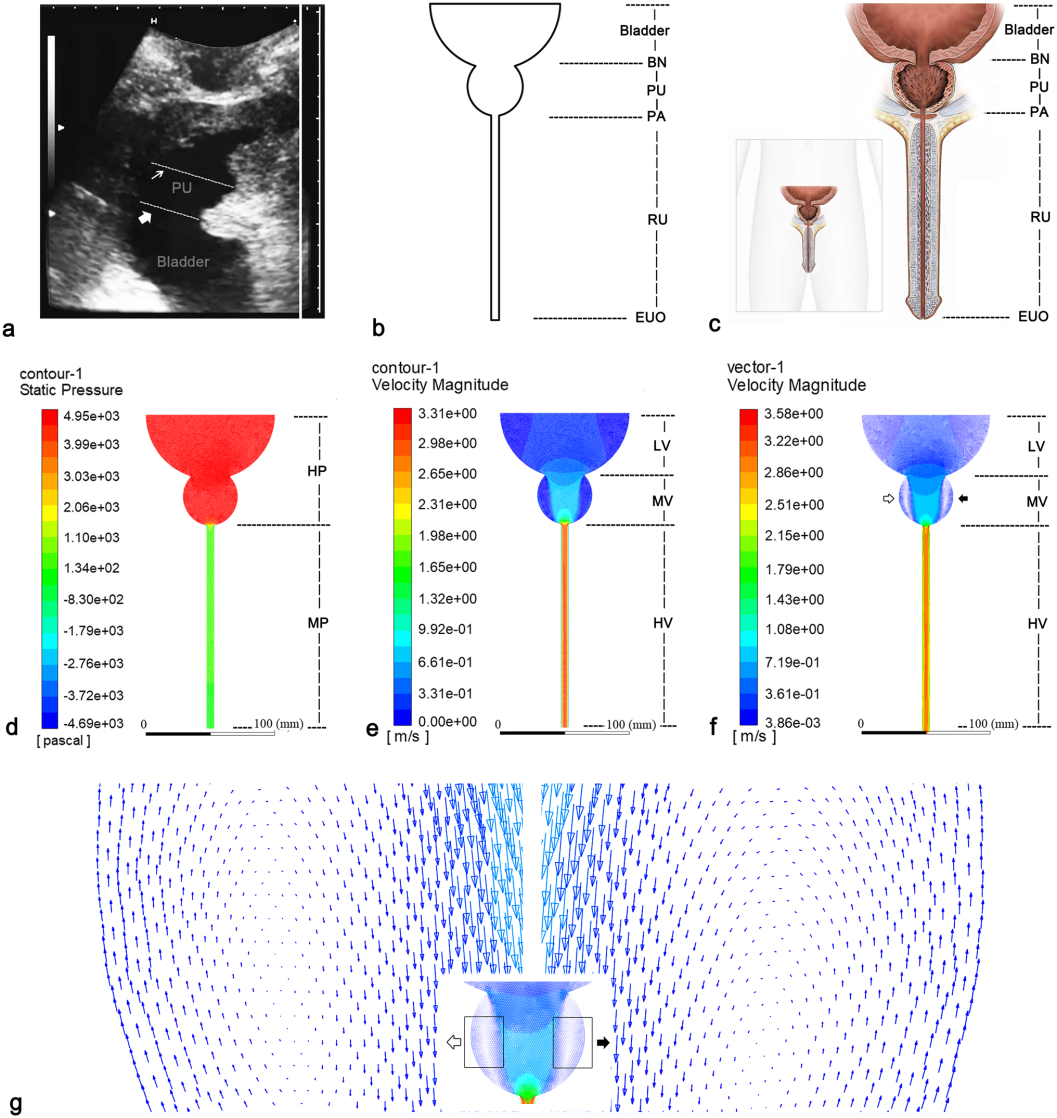
Standardized and idealized two-dimensional CFD model after transurethral surgery (CATS-1st). (**a**) The anteroposterior diameters of BN (broad arrow) and PU (narrow arrow) from sagittal ultrasound. (**b**) Geometric model of CATS-1st. (**c**) The schematic diagram of the surgical scenario corresponding to the geometric model (front view). (**d**) Stress contour of CATS-1st. (**e**) Velocity contour of CATS-1st. (**f**) Velocity vector diagram of CATS-1st. Vorticesare showed on right (white arrow) and left (black arrow) side in the PU. (**g**) Magnified votices on right (white arrow) and left (black arrow) side in the PU. *BN* bladder neck, *CFD* computational fluid dynamics, *EUO* external urethral orifice, *HP* high pressure, *HV* high velocity, *LD-PU* longitudinal diameter of prostatic urethra, *LV* low velocity, *MP* medium pressure, *MV* medium velocity, *PA* prostate apex, *PU* prostatic urethra, *RU* the rest of the urethra.

Ultrasound images of the bladder and the PU were obtained from a 79-year-old male patient who had undergone the ultrasound examination 1 month after his transurethral thulium laser enucleation of the prostate (ThuLEP) at the First Hospital of Shanxi Medical University. At the time of examination, the patient [pre-operative prostate volume: 78.6 cm^3^, body mass index (BMI): 25.7 kg/m^2^] had no complications, including urethral stricture, urinary incontinence, and urinary tract infection. The patient was asked to perform spontaneous urinary voiding in a standing position, during, which dynamic images were recorded using transperineal ultrasound (Hitachi, Japan, EUB-7000) in maximum sagittal view, while the urine flow rate was simultaneously monitored using a urine dynamic analysis system (Laborie, Canada, ULC/Triton). When the maximum urine flow rate (Qmax) appeared, the anteroposterior diameters were measured using ultrasound, for both bladder neck (BN, 3.0 cm) and PU (4.2 cm) (Fig. 1a). The participant had provided his consent for the ultrasound examination and the storage of resultant data for research purposes. The study protocol was approved by the Institutional Ethics Board of First Hospital, Shanxi Medical University, and the whole study was performed in accordance with the relevant guidelines/regulations.

### Geometric modeling

In order to conduct simulation experiments, a standardized and idealized geometric two-dimensional CFD model after transurethral surgery (CATS-1st) was developed for post-surgery mid-voiding.

In order to focus the investigation on the impact of different diameters of PU, first of all, the important structures of the lower urinary tract in elderly men were simplified for clear understanding. First, the lower urinary tract was supposed to comprise the lower half of the bladder, the PU and the rest of the urethra (RU). Accordingly, the three of them were assumed to be near-hemispheric, near-spherical, and long cylindrical in space, respectively. The PU was bound to the prostate apex at the bottom, and to the bladder neck (BN), which was simplified without thickness, at the top. Next, the diameters of the lower half of the bladder and the RU were determined to be 10.0 cm and 0.6 cm, respectively^10^. On the basis of the diameters measured mid-voiding using ultrasound and the parameters obtained from the published literature^11,12^, the longitudinal diameter of PU (LD-PU), the transverse diameter of BN (TD-BN), the transverse diameter of PU (TD-PU), and the length of RU were determined to be 3.8 cm, 3.0 cm, 4.2 cm, and 15.7 cm^10^, respectively. LD-PU was defined as the distance between the midpoint of BN and the midpoint of the prostate apex. Finally, the bending angles of both PU and RU toward the anterior direction were deliberately ignored.

On the basis of the simplified anatomical structure of the lower urinary tract, CATS-1st was developed to reflect the structural characteristics of the maximum coronal profile of the corresponding lumen (Fig. 1b,c). The model was drafted using AutoCAD 2016 software (Autodesk Inc., San Rafael, CA, USA).

### Mesh modeling

After geometric modeling, the developed model CATS-1st was meshed with triangle elements in a Cartesian coordinate system using the analysis software ICEM CFD 19.0 (Ansys Inc., USA). The line segments at the top and bottom of the model were designated as the inlet and outlet, respectively, while the rest of the contour curves were specified as walls.

Model construction was performed using different grid sizes of 13, 18, 25, and 35 thousand mesh elements, and the urinary flow field data under each grid size was obtained. Subsequently, the midpoint velocities of the external urethral orifice (MV-EUO) and the transverse diameter of the vortex (TD-V, mean of both side values) were compared. The grid independence evaluation revealed that there was little difference (< 2%) in the calculation results with the increase in the number of grids beyond the grid size of 18 thousand elements. Therefore, a triangle unstructured mesh with a grid size greater than 18 thousand elements and smaller than 35 thousand elements was selected as CATS-1st (25,372 mesh elements) for the CFD simulations. No boundary-fitted prism layer was adopted as it was considered that the grid division precision was sufficient to simulate the boundary layer and that the prismatic layer exerted a negative impact on the overall mesh quality due to the narrow drainage basin at the urethra.

### CFD simulations

The CFD simulation was performed over the computational flow domain using Ansys Fluent 19.0 (Ansys Inc., USA). The model was developed for a grid body with all structures having no elasticity. The working fluid was assumed to be steady, homogeneous, incompressible, adiabatic, and Newtonian^13^, and having a urinary density of 1035 kg/m^3^ and dynamic viscosity of 0.8583 × 10^–3^ kg/m-s (Pa s) at 37 ℃ as reported in the literature^14^. Assuming the intra-abdominal pressure (Pabd) of 1961.3 Pa (20.0 cmH_2_O), the inlet pressure was set to 4958.8 Pa (50.6 cmH_2_O) in accordance with the previously reported estimated intravesical pressure (Pves)^15^, while the outlet pressure was set to 0 Pa. The urinary viscosity was supposed to vary inappreciably with the physiological shear rate and was set to 0 mL per second for the wall fitting with no slip assumption.

Since previous experimental studies have evidenced the occurrence of the vortex in the PU both prior to or during surgery^3,4^, the Reynolds averaged Navier–Stokes equation (RNG k-epsilon turbulence model) was applied to simulate the urine flow status. A second-order pressure discretization scheme was applied for pressure calculations, while a second-order upwind scheme was utilized for momentum and turbulence transport equations. A simple-consistent (SIMPLEC) algorithm was applied to resolve the pressure–velocity coupling problem. The turbulent intensity was set to 5%.

The pressure and velocity in CATS-1st were monitored through flow dynamics simulation, and the MV-EUO, the TD-V, and the diameter ratio of PU (RPU-1, calculated from TD-PU/TD-BN; RPU-2, calculated from RPU-1/LD-PU) were calculated. In addition, the relationships between TD-V and RPU-1, TD-V and RPU-2, MV-EUO and RPU-1, and MV-EUO and RPU-2 were determined.

### Quantitative amplification

In consideration of the different shapes of PU in the patients after different transurethral surgeries, an additional set of 209 examples was quantitatively amplified based on CATS-1st. The amplification was performed according to a three-dimensional array of size [3][5][14], which comprised three groups of LD-PU (I: 3.6 cm; II: 3.8 cm; III: 4.0 cm), each containing five subgroups of TD-BN (A: 2.6 cm; B: 2.8 cm; C: 3.0 cm; D: 3.2 cm; E: 3.4 cm), while each subgroup contained 14 examples of TD-PU (every 0.2 cm in the range of 2.0–4.6 cm).

All the amplified examples were processed using flow dynamics simulation followed by CATS-1st simulation.

### Statistical analysis

All the data were analyzed statistically using the Statistical Package for Social Sciences software v.19.0 (SPSS Inc., Chicago, IL, USA). Medians and interquartile ranges (IQR) were determined for the continuous variables. Mann–Whitney U test was performed to compare the means between vortex and non-vortex examples. A p value of less than 0.05 was considered significant.

### Ethical approval

The study was approved by the institutional ethics board of First Hospital of Shanxi Medical University.

### Informed consent

Written informed consent was obtained from the patient for the publication of this study and any accompanying images.

## Results

The present study concerns the modeling and simulation of 210 simplified CATS examples, including CATS-1st and 209 additional amplified examples.

Figure 1 presents the structure, pressure, and velocity values for the CATS-1st examples. There is high pressure in both bladder and PU, and a medium or low pressure in RU (Fig. 1c). High, medium, and low velocities are displayed in RU, PU, and bladder, respectively. Rapid velocity changes are concentrated in and around PU (Fig. 1d), While the laminar flow is present in all the examples. A few examples within each subgroup presented vortex on both the sides of PU. In regard to CATS-1st, a vortex appeared on both the sides of PU with mean transverse and longitudinal diameters of 1.1 cm and 3.0 cm, respectively (Fig. 1e,f, respectively).

**Figure 2:**
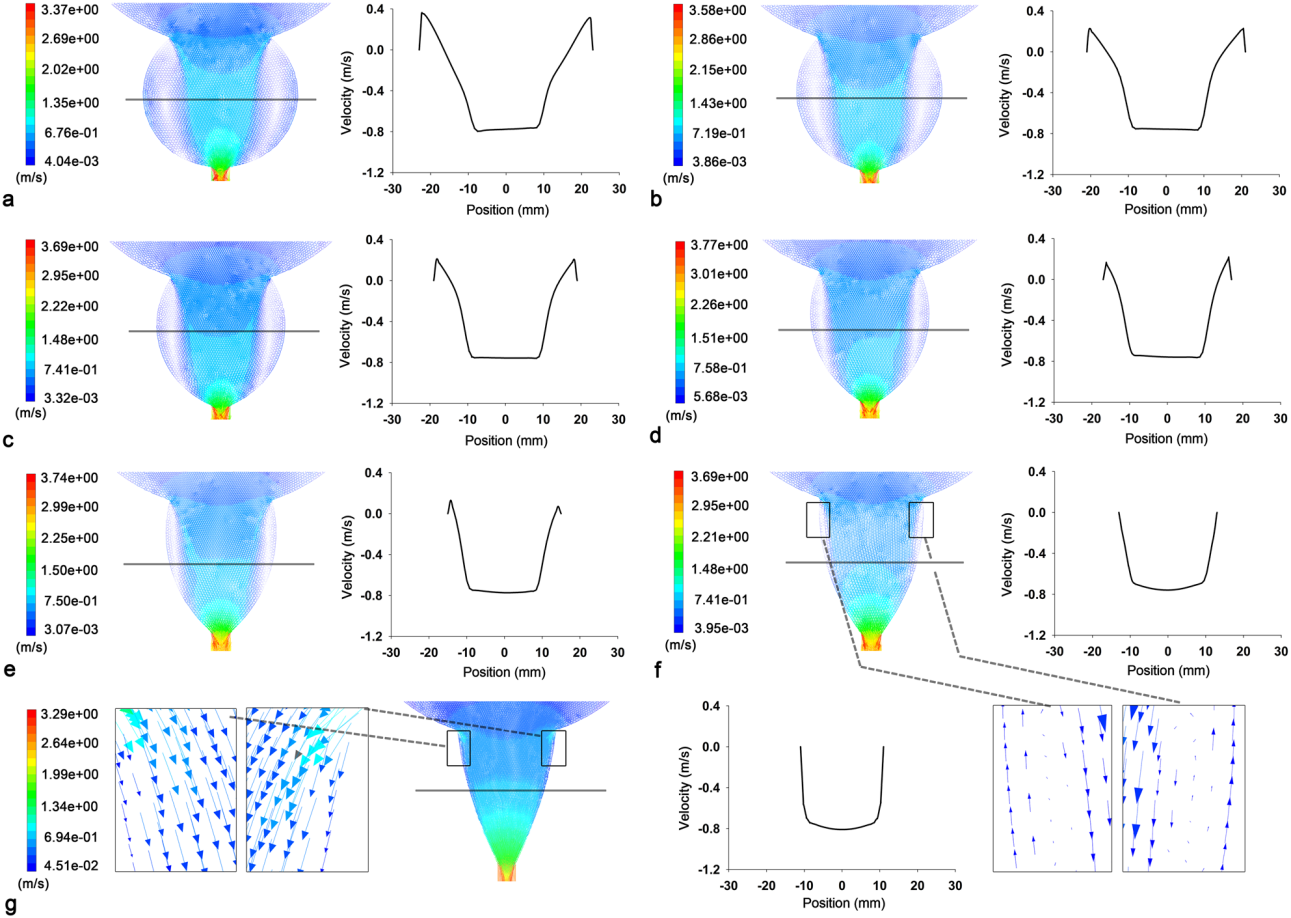
Velocity vector diagrams and velocity/position graphs of several examples. Examples of the subgroup have the same LD-PU (3.8 cm) and TD-BN (3.0 cm), but each of them has different TD-PU, which is 4.6 cm (**a**), 4.2 cm ((**b**), CATS-1st), 3.8 cm (**c**), 3.4 cm (**d**), 3.0 cm (**e**), 2.6 cm (**f**), and 2.2 cm (**g**), respectively. Compared with the velocity/position graph (black and white), the velocity vector diagrams (color) show the vortex better due to the magnification function. No vortex presents in the example with 2.2 cm (**g**) in the TD-PU. *CATS-1st* standardized and idealized two dimensional computational fluid dynamics model after transurethral surgery, *LD-PU* longitudinal diameter of prostatic urethra, *TD-BN* transverse diameter of the bladder neck, *TD-PU* transverse diameter of the prostatic urethra.

Figure 2 presents the changes in the vortices in the PU of the subgroup containing CATS-1st belongs (LD-PU = 3.8 cm, TD-BN = 3.0 cm); it was observed that TD-V gradually decreased with reducing TD-PU until the vortex disappeared. The velocity/location graphs of the vortex examples reveal that the downward velocities gradually decreased to 0 m/s from the transverse center to both the sides, then went into the opposite direction, and finally declined back to 0 m/s when they reached the non-slip boundary layer.

**Figure 3:**
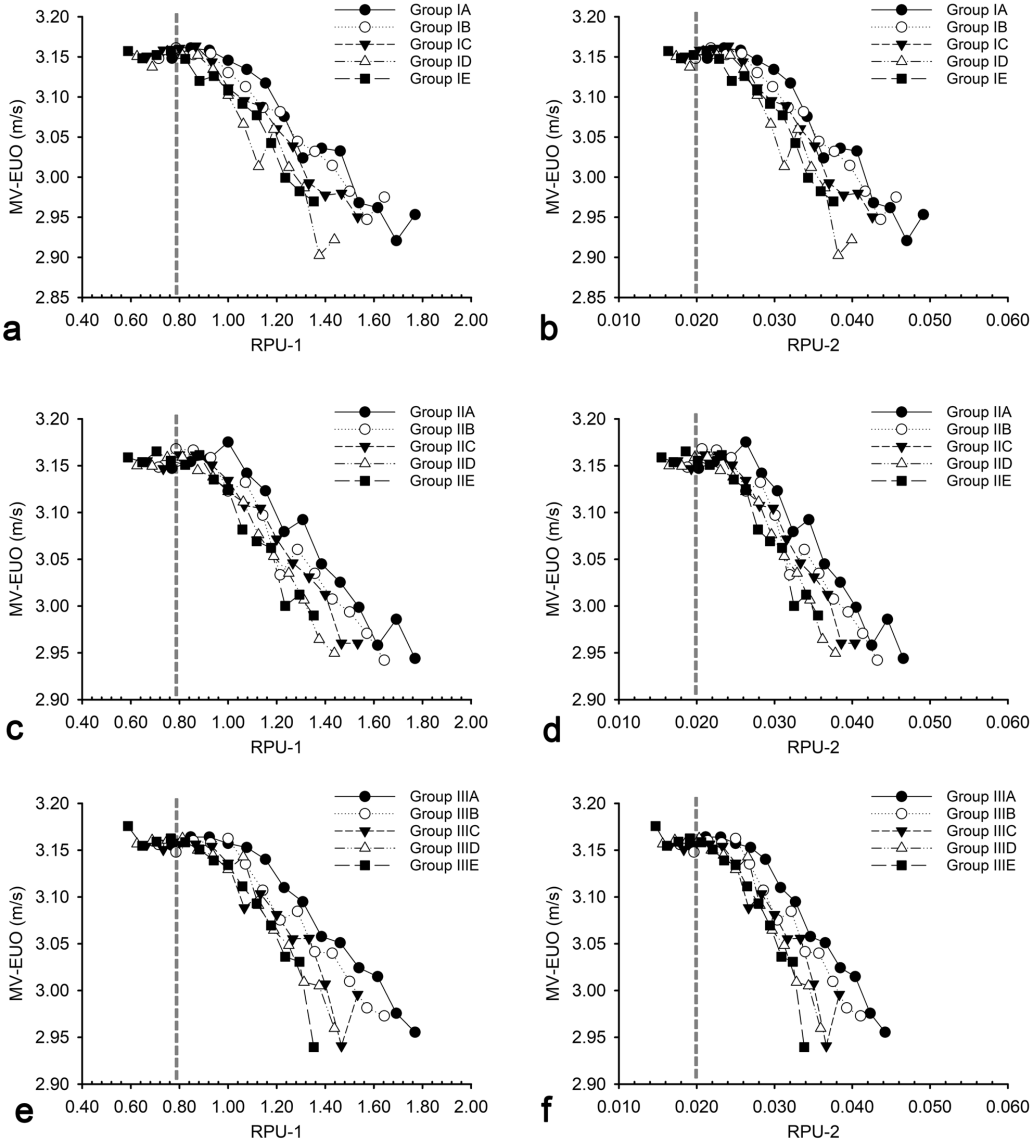
Relationships between MV-EUO and RPU in groups (210 examples). The LD-PU in group I, II and III is 3.6 cm, 3.8 cm, and 4.0 cm, respectively. MV-EUOs increase gradually by reducing RPU-1 and RPU-2. The abscissas of the dotted line are 0.79 in RPU-1 and 0.02 in RPU-2, respectively. *LD-PU* longitudinal diameter of prostatic urethra, *MV-EUO* midpoint velocity of the external urethral orifice, *RPU* diameter ratio of prostatic urethra, *RPU-1* the first diameter ratio of prostatic urethra, *RPU-2* the second diameter ratio of prostatic urethra.

Figure 3 presents the variations in MV-EUO with RPU-1 and RPU-2 for all the 210 examples. In all groups, MV-EUO increased gradually by reducing RPU-1 and RPU-2, and finally stabilized while they approached 0.79 and 0.02, respectively. The relatively stable value of MV-EUO across the groups was concentrated and located in the region of a high velocity of over 3.16 m/s (IQR 3.15–3.16).

**Figure 4:**
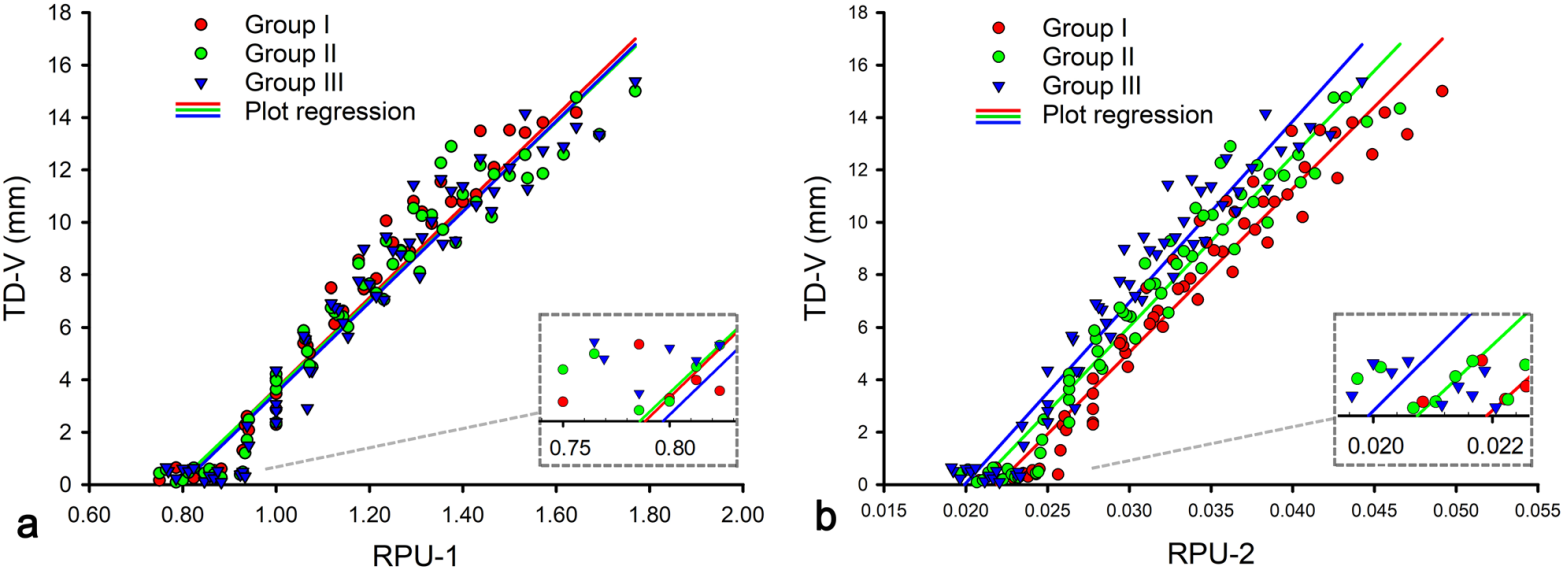
Scatterplots displaying regression line of TD-V with RPU in vortex examples (182 examples). The LD-PU in group I, II and III is 3.6 cm, 3.8 cm, and 4.0 cm, respectively. *LD-PU* longitudinal diameter of prostatic urethra, *PU* prostatic urethra, *RPU* diameter ratio of prostatic urethra, *RPU-1* the first diameter ratio of prostatic urethra, *RPU-2* the second diameter ratio of prostatic urethra, *TD-V* the transverse diameter of the vortex.

Figure 4 presents the relationship between TD-V and RPU for the 182 vortex examples across all groups. The TD-V declined linearly with decreasing RPU-1 and RPU-2, while the minimum RPU-1 and RPU-2 of these vortex examples reached values close to 0.79 and 0.02, respectively.

Table 1 lists the characteristics of the CATSs in the groups. As visible, a significant (*p* < 0.01) decrease in MV-EUO was observed for the vortex examples.

**Table 1:** Characteristics of the examples in groups (210 examples).

	Group-I	Group-II	Group-III	All
LD-PU (cm)	3.6	3.8	4.0	
CATS models	70 (33.33%)	70 (33.33%)	70 (33.33%)	210 (100.0%)
Vortex models	60 (85.71%)	61 (87.14%	61 (87.14%	182 (86.67%)
Non-vortex models	10 (14.29%)	9 (12.86%)	9 (12.86%)	28 (13.34%)
TD-V (mm)	7.26 (2.31, 10.69)	6.58 (2.03, 10.41)	6.92 (1.87, 10.55)	6.85 (2.28, 10.46)
RPU-1 of vortex models	1.17 (0.96, 1.37)	1.15 (0.94, 1.37)	1.15 (0.94, 1.37)	1.15 (0.94, 1.36)
RPU-1 of non-vortex models	0.70 (0.64, 0.74)	0.69 (0.64, 0.72)	0.69 (0.64, 0.72)	0.69 (0.65, 0.73)
RPU-2 of vortex models	0.032 (0.027, 0.038)	0.031 (0.025,0.036 )	0.029 (0.024, 0.034)	0.031 (0.025, 0.036)
RPU-2 of non-vortex models	0.019 (0.018, 0.021)	0.018 (0.017, 0.019)	0.017 (0.016, 0.018)	0.018 (0.017, 0.019)
MV-EUO of vortex models(m/s)	3.07 (2.99, 3.13)	3.08 (3.01, 3.14)	3.09 (3.03, 3.15)	3.08 (3.01, 3.14)
MV-EUO of non-vortex models(m/s)	3.15 (3.15, 3.16)*	3.15 (3.15, 3.16)*	3.16 (3.16, 3.16)*	3.15 (3.15, 3.16)*

*LD-PU* longitudinal diameter of prostatic urethra, *MV-EUO* midpoint velocity of the external urethral orifice, *RPU-1* the first diameter ratio of prostatic urethra, *RPU-2* the second diameter ratio of prostatic urethra, *TD-V* the transverse diameter of the vortex.

*Comparing with the non-vortex examples, a significant decrease in MV-EUO was observed for the vortex examples: p < 0.001 (group-I), p = 0.002 (group-II), p < 0.001 (group-III), p < 0.001 (all).

## Discussion

Most of the transurethral surgeries including transurethral resection, enucleation, dilation and vaporization of the prostate (TU-RP, TU-EP, TU-DP, and TU-VP, respectively) are aimed at relieving the obstruction in PU. It is possible to relieve urinary tract obstruction even when no more than half of the total tissue area is resected. This is convincing evidence that restoring the PU lumen is more important than removing the proliferative tissues. Despite being considered effective treatments, the surgical approaches to relieving obstruction have drawn a mixed response from a clinical study^16^. We hold that relieving the obstruction in PU depends not just on expanding the volume, and rather on restoring the luminal shape to become consistent with the urodynamic requirements. Moreover, studies have confirmed that the vortex occurs in the narrow lumen^17^ as well as in the completely-hollowed cavity^18^ with a boost of laminar flow. It is entirely possible that the fluid energy loss caused by the vortex^2^ affects urination. Therefore, greater attention is warranted for analyzing the urine flow in the hollowed PU after transurethral surgery. However, so far, only a few studies have focused on this research area, and to the best of our knowledge, the present primary research is the first attempt to model the effects of PU after transurethral surgery for BPH using CFD.

As a consequence of the fundamental disparities among the selected operative methods or in the skills of the surgeons, and because of the individual differences in the BPH, the patients present different shapes of prostatic urethra (PU) after their transurethral surgeries. The PU might be broader in the TU-EP for TD-PU and wider after the removal of the hyperplasia tissue from the bladder neck for TD-BN. It might also be longer in the severe prostatic hyperplasia tissue after surgery for LD-PU. As there is no consensus on the normative standards for the quantification of surgical completion, different idealized geometries were created, not just for one particular surgical scenario, rather for many possible and representative scenarios.

In order to acquire direct observations of the urine flow dynamics in the PU, a practical examination is necessary. As direct evaluation using the current invasive and non-invasive urodynamics measurement tools proves to be difficult and inadequate^6^, the technical field of CFD emerges as a better approach to analyze the physiological and pathological functions occurring inside tubular organs^10,19,20^. Since rapid results with adequate accuracy are expected from the CFD models, in the present study, rather than developing overcomplicated and slow-calculating models^21^, the PU was drafted as a near-spherical structure truncated by two parallel sections (the planes of BN and prostate apex), and a feasible two-dimensional model was designed first instead of an aggressive three-dimensional model, which was based on model simplification and anatomical knowledge.

According to the distribution of pressure and velocity, different variation characteristics were observed in the lower urinary tract. First, the pressure decreased rapidly between the PU and the RU, compared to the pressure between bladder and PU, which might be related to the faster contraction at the prostatic apex compared to a relatively open BN. Second, the direction of velocity was not always toward the urethral outlet. Using the velocity/position graphs and the enlarged velocity vector diagrams, it was determined that vortices were present on both sides of the PU, while laminar flow and vortex flow coexisted in most of the examples. Third, the velocity accelerated rapidly around the PU, which was the region where the shape contracted twice and expanded once. Within each group, the vortex examples presented lower MV-EUO compared to the non-vortex examples. Moreover, the MV-EUO gradually recovered with the decreasing RPU until the vortex disappeared. The recovering peak flow in the vortex examples could be attributed to the disappearance of the vortex, which supported the hypothesis of vortex dissipation to fluid energy.

In each subgroup, with reducing TD-PU, the vortices on either side of the PU shrank gradually until disappearance. In other words, the closer the PU was to the trapezoid, the fewer were the vortices it exhibited. This occurred because the shape of the PU is determined, in general, by four factors, namely, LD-PU, TD-BN, TD-PU and the transverse diameter of RU. Since, in the present study, the transverse diameter of RU was maintained constant (0.6 cm), the vortex was correlated positively with TD-PU and negatively with TD-BN and LD-PU. RPU-1 and RPU-2 might serve as suitable predictors of the presence of vortex, and this hypothesis was also confirmed in the present study. According to our knowledge, the present novel primary research is a pioneer in concurrently evaluating all these three features/characteristics.

Furthermore, with reducing RPU-1 and RPU-2, there was a linear decline in TD-V until it disappeared. Although the minimum RPU in the vortex examples was not identical to the corresponding values in the other three groups, all these values corresponded closely to each other. In regard to TD-V, RPU-2 exhibited better regularity compared to RPU-1. Moreover, MV-EUO increased gradually with decreasing RPU-1 and RPU-2 in all the groups, and finally stabilized at a high-velocity level. This was a reminder of the fluid energy loss being gradually reduced and the MV-EUO being gradually restored with the shrinking vortex. Furthermore, MV-EUO was affected minutely by the vortex when the latter was reaching disappearance. In this context, it is noteworthy that, even though the minimum RPU of the vortex examples is one of the sensitive boundaries reflecting the physical condition of the vortex, it does not represent a boundary in clinical outcomes. Therefore, clinical observation and correlation analysis are nonetheless required for determining the minimum clinical RPU of the vortex.

The present study also had certain limitations. First, due to a lack of well-recognized diameters of PU after transurethral surgery^8^, only one patient’s diameters could be selected for modeling. A larger dataset is obviously recommended. And, it would be better to obtain the other parameters from the corresponding patients rather than from retrospective studies. The future scope of the present research includes designing further realistic models and confirming the relationship between RPU and post-void residual volume with a larger population. Second, a three-dimensional model clearly should first be considered before extrapolating conclusions to surgical scenario. However, considering that it is difficult to make a balance between keeping a model simple to allow for computation versus a model with so much complexity that errors easily propagate, the whole urethra was simplified as a two-dimensional model for first application of CFD. Third, since there is no consensus among the researchers on the most suitable non-invasive method for monitoring the urinary flow in BPH disease, CFD could not be compared to a gold standard. Furthermore, it is not possible to measure the parameters of urine velocity, bladder pressure, and PU diameters during urination in real-time at multiple locations simultaneously in the bladder and the urethra, rendering a clinical validation challenging. Despite these limitations, the non-invasive visualization of the intra-urethral vortices^22^ in the PU would enable the urologists to reconsider the technical standard of prostate resection and consequently realize a further precise surgical method for the BPH followed by voiding dysfunction in elderly males. The findings of the present study represent a significant step in the direction of investigating the urine flow behavior within the PU after transurethral surgery for BPH.

The present study attempted to explore the effects of different diameter ratios of PU on the mechanical behavior of urine flow mid-voiding after transurethral surgery. The CFD model (CATS) revealed that rapid changes in the pressure and velocity were concentrated in and around the PU, a region that presents huge shape changes after the surgery. Moreover, with decreasing RPU-1 and RPU-2, the vortices on either side of the PU shrank linearly, while the MV-EUO recovered gradually. In regard to urethral vortex flow, CFD is currently a viable research tool having a potential for high clinical impact.

## Abbreviations

BN: Bladder neck
BPH: Benign prostatic hyperplasia
CATS: Two-dimensional CFD model after transurethral surgery
CATS-1st: Standardized and simplified two-dimensional CFD model after transurethral surgery
CFD: Computational fluid dynamics
LD-PU: The longitudinal diameters of prostatic urethra
MV-EUO: The midpoint velocity of the external urethral orifice
PU: Prostatic urethra
RPU: Ratio of prostatic urethra
RPU-1: The first diameter ratio of prostatic urethra
RPU-2: The second diameter ratio of prostatic urethra
RU: The rest of the urethra
TD-BN: The transverse diameters of the bladder neck
TD-PU: The transverse diameters of the prostatic urethra
TD-V: The transverse diameter of the vortex

## References

[1] Rademakers K, Drake MJ, Gammie A, Djurhuus JC, Rosier PFWM, Abrams P, Harding C (2017). Male bladder outlet obstruction: Time to re-evaluate the definition and reconsider our diagnostic pathway? ICI-RS 2015. Neurourol. Urodyn..

[2] Ishii T, Kambara Y, Yamanishi T, Naya Y, Igarashi T (2014). Urine flow dynamics through prostatic urethra with tubular organ modeling using endoscopic imagery. IEEE J. Transl. Eng. Health Med..

[3] Minagawa T, Tezuka M, Ogawa T, Ishizuka O (2020). Vorticity in lower urinary tract can be assessed and associates with urinary tract morphology in men. Neurourol. Urodyn..

[4] Minagawa T, Ogawa T, Ishizuka O (2019). Fluid dynamic assessment of the lower urinary tract: Exploratory research to observe vorticity in the prostatic urethra after transurethral enucleation. Int. J. Urol..

[5] Presicce F, Nunzio C, Gacci M, Finazzi Agrò E, Tubaro A (2017). Non-invasive ultrasound measurements in male patients with LUTS and benign prostatic obstruction: Implication for diagnosis and treatment. Minerva Urol. Nefrol..

[6] Guneyli S, Ward E, Thomas S, Yousuf AN, Trilisky I, Peng Y, Antic T, Oto A (2016). Magnetic resonance imaging of benign prostatic hyperplasia. Diagn. Interv. Radiol..

[7] Cohen AJ, Baradaran N, Mena J, Krsmanovich D, Breyer BN (2019). Computational fluid dynamic modeling of urethral strictures. J. Urol..

[8] Ishii T, Ho CK, Nahas H, Yiu BYS, Chee AJY, Yu ACH (2019). Deformable phantoms of the prostatic urinary tract for urodynamic investigations. Med. Phys..

[9] Glemain P, Buzelin JM, Cordonnier JP (1993). New urodynamic model to explain micturition disorders in benign prostatic hyperplasia patients. Pressure-flow relationships in collapsable tubes, hydraulic analysis of the urethra and evaluation of urethral resistance. Eur. Urol..

[10] Eichaker L, Li C, King N, Pepper V, Best C, Onwuka E, Heuer E, Zhao K, Grischkan J, Breuer C, Johnson J, Chiang T (2018). Quantification of tissue-engineered trachea performance with computational fluid dynamics. Laryngoscope.

[11] Agrawal V, Khullar R, Jha AK (2020). Assessment of posterior urethra in benign prostatic hyperplasia and after its surgery. Urol. Ann..

[12] Watanabe H, Takahashi S, Ukimura O (2014). Urethra actively opens from the very beginning of micturition: A new concept of urethral function. Int. J. Urol..

[13] Kren J, Horák M, Zát'ura F, Rosenberg M (2001). Mathematical model of the male urinary tract. Biomed. Pap. Med. Fac. Univ. Palacky Olomouc Czech Repub..

[14] Inman BA, Etienne W, Rubin R, Owusu RA, Oliveira TR, Rodriques DB, Maccarini PF, Stauffer PR, Mashal A, Dewhirst MW (2013). The impact of temperature and urinary constituents on urine viscosity and its relevance to bladder hyperthermia treatment. Int. J. Hypertherm..

[15] Chen SF, Lee CL, Kuo HC (2019). Change of detrusor contractility in patients with and without bladder outlet obstruction at ten or more years of follow-up. Sci. Rep..

[16] Cornu JN, Ahyai S, Bachmann A, de la Rosette J, Gilling P, Gratzke C, McVary K, Novara G, Woo H, Madersbacher S (2015). A systematic review and meta-analysis of functional outcomes and complications following transurethral procedures for lower urinary tract symptoms resulting from benign prostatic obstruction: An update. Eur. Urol..

[17] Pel JJ, van Mastrigt R (2007). Development of a CFD urethral model to study flow-generated vortices under different conditions of prostatic obstruction. Physiol. Meas..

[18] Wheeler AP, Morad S, Buchholz N, Knight MM (2012). The shape of the urine stream—From biophysics to diagnostics. PLoS ONE.

[19] Ohnishi K, Okihara K, Tanaka T, Watanabe M, Hayami H, Ohe H, Watanabe H, Yokobori T, Sasaki S (1991). A study of the simulation model of the lower urinary tract for urodynamics–(the first report)—Theoretical evaluation of hydrodynamic model. Hinyokika Kiyo..

[20] Wong KK, Wang D, Ko JK, Mazumdar J, Le TT, Ghista D (2017). Computational medical imaging and hemodynamics framework for functional analysis and assessment of cardiovascular structures. Biomed. Eng. Online.

[21] Morris PD, Narracott A, von Tengg-Kobligk H, Silva Soto DA, Hsiao S, Lungu A, Evans P, Bressloff NW, Lawford PV, Hose DR, Gunn JP (2016). Computational fluid dynamics modelling in cardiovascular medicine. Heart.

[22] Ishii T, Yiu BYS, Yu ACH (2017). Vector flow visualization of urinary flow dynamics in a bladder outlet obstruction model. Ultrasound Med. Biol..

